# Predicting the Mechanical Response of Polyhydroxyalkanoate Biopolymers Using Molecular Dynamics Simulations

**DOI:** 10.3390/polym14020345

**Published:** 2022-01-17

**Authors:** Karteek K. Bejagam, Nevin S. Gupta, Kwan-Soo Lee, Carl N. Iverson, Babetta L. Marrone, Ghanshyam Pilania

**Affiliations:** 1 Los Alamos National Laboratory, Materials Science and Technology Division, Los Alamos, NM 87545, USA; karteekbeja@lanl.gov; 2 Los Alamos National Laboratory, Chemistry Division, Los Alamos, NM 87545, USA; nevin5@lanl.gov (N.S.G.); kslee@lanl.gov (K.-S.L.); iverson@lanl.gov (C.N.I.); 3 Los Alamos National Laboratory, Bioscience Division, Los Alamos, NM 87545, USA; blm@lanl.gov

**Keywords:** PHAs, atomistic simulations, polymer design, property predictions, chemical trends

## Abstract

Polyhydroxyalkanoates (PHAs) have emerged as a promising class of biosynthesizable, biocompatible, and biodegradable polymers to replace petroleum-based plastics for addressing the global plastic pollution problem. Although PHAs offer a wide range of chemical diversity, the structure–property relationships in this class of polymers remain poorly established. In particular, the available experimental data on the mechanical properties is scarce. In this contribution, we have used molecular dynamics simulations employing a recently developed forcefield to predict chemical trends in mechanical properties of PHAs. Specifically, we make predictions for Young’s modulus, and yield stress for a wide range of PHAs that exhibit varying lengths of backbone and side chains as well as different side chain functional groups. Deformation simulations were performed at six different strain rates and six different temperatures to elucidate their influence on the mechanical properties. Our results indicate that Young’s modulus and yield stress decrease systematically with increase in the number of carbon atoms in the side chain as well as in the polymer backbone. In addition, we find that the mechanical properties were strongly correlated with the chemical nature of the functional group. The functional groups that enhance the interchain interactions lead to an enhancement in both the Young’s modulus and yield stress. Finally, we applied the developed methodology to study composition-dependence of the mechanical properties for a selected set of binary and ternary copolymers. Overall, our work not only provides insights into rational design rules for tailoring mechanical properties in PHAs, but also opens up avenues for future high throughput atomistic simulation studies geared towards identifying functional PHA polymer candidates for targeted applications.

## 1. Introduction

The plastics industry has revolutionized our modern society and it is hard to imagine the world around us without plastics. Nevertheless, petroleum-based synthetic plastics are designed for performance and durability, but not for biodegradability and recyclability [1]. Moreover, single-use plastics constitute 40% of the total plastics and the majority of them end up in landfills and oceans causing a severe burden to the environment [2,3,4,5]. In recent years, there has been a huge interest in replacing conventional plastics with biodegradable functional polymers for a sustainable and greener future [6,7]. Polyhydroxyalkanoates (PHAs), representing one such promising class of polyesters, have recently gained significant attention due to their unique features such as biodegradability and chemical diversity [8,9,10,11].

PHAs are naturally occurring biopolymers synthesized by several microorganisms as energy reserve and carbon storage molecules using sunlight, water, and atmospheric carbon dioxide [12,13,14]. Additionally, there are also well established chemical routes for controlled PHA synthesis [15,16,17,18,19]. Given that over a hundred PHA monomers are synthetically known, the chemical space spanned by this class of materials is rather vast. The monomers can further be combined, taken two or more at a time, to form multi-component copolymers or polymer blends. Furthermore, these multi-component systems can be generated at different compositions (ratio of different monomers) as well as configurations (relative position of different monomers across the polymer backbone), which further broadens the chemical design space. A synthesis strategy solely based on chemical-intuition is unlikely to be efficient in exploring such a large chemical space, in order to design a polymer for a specific application. For this reason, more efficient and predictive methods based on state-of-the-art computational approaches that enable accurate property predictions for a better understanding of quantitative structure–property relationships (QSPR) in PHA chemical space are desired.

Often poor mechanical properties associated with the commonly occurring PHAs, such as poly(3-hydroxybutyrate) or P3HB, limit their utilization for packaging applications [20,21]. The poor mechanical properties lead to poor flexibility and strength, and high brittleness for the biopolymers, limiting their processability and end-use applications [7]. Therefore, as a specific challenge, one needs to find PHA polymer motifs and compositions that exhibit superior mechanical properties, allowing them as a viable alternative of conventional packaging materials [20]. Tailoring the mechanical properties can be achieved either by combining monomers with diverse chemistries to form either copolymers or blends [22,23,24,25] or by adding plasticizers to the polymer matrix [26,27]. To accelerate the rational design of PHA-based polymers, the chemical design space has to be efficiently explored and the structure–property relationships established to identify new polymers with enhanced properties [28].

Molecular dynamics (MD) simulations using physics-based models have been proven highly efficient in predicting a range of polymer properties [29,30,31,32]. MD simulations were used in studying the miscibility of PHA and polylactide (PLA) polymers [33] and have also been employed in studying the mechanical properties of linear polymers [34], branched polymers [35], crosslinking polymers [36], and even the properties of the graphene-polymer interface [37,38]. In our previous work [29], we developed a modified polymer consistent force field (mPCFF) to represent PHA-based polymers by refining the torsional potentials associated with the polymer backbone based on density functional theory (DFT) calculations. These parameters were employed to represent the underlying potential energy surface for PHA molecules. We employed MD simulations to predict the glass transition temperature ($T_{g}$) of PHA homopolymers [29] and also systematically explored the compositional- and configurational-dependence of $T_{g}$ in binary copolymers and blends of PHA polymers [30]. In this contribution, we extend our past work to study the mechanical properties of PHA-based polymers. In the present work, MD simulations are employed to determine the mechanical properties; specifically, Young’s modulus and yield stress, by applying strain at different rates as well as at different temperatures. In addition, we explore the influence of polymer structure, such as length of the side chain and polymer backbone, and also the chemical nature of the side chain functional group on the mechanical properties to understand systematic design rules in the underlying chemical space.

## 2. Materials and Methods

Classical MD simulations were performed using large-scale atomic/molecular massively parallel simulator (LAMMPS) 30 April 2019 version [39]. The initial configurations of an amorphous polymer box consisting of 10 chains, each with 500 monomer units, were generated using Enhanced Monte Carlo (EMC) code, (version 9.4.4) at 1 gm/cm^3^ [40]. Customized mPCFF parameters [29] were adopted to represent PHA molecules, which have been developed by modifying the torsional potentials in the polymer backbone based on DFT computations. All of the PHA monomeric units were generated with a *R*-chiral center, consistent with experimental observations [15,41]. The chemical structures of repeating units of all the polymers simulated in this study are shown in Figure S1 of the Supplementary Materials. Equations of motion were integrated with a time-step of 1 fs and the velocity-Verlet algorithm [42] was used to update the positions and velocities at each time-step. In all the simulations, three-dimensional periodic boundary conditions were applied. Temperature and pressure were controlled by employing the Nosé–Hoover thermostat and barostat, respectively [43,44]. A cutoff of 9.5 Å was used for treating real space interactions. Long-range Coulombic interactions were treated using the particle-particle particle-mesh (PPPM) method [45] and long-range corrections to the pressure and energy terms were included.

**Figure 1:**
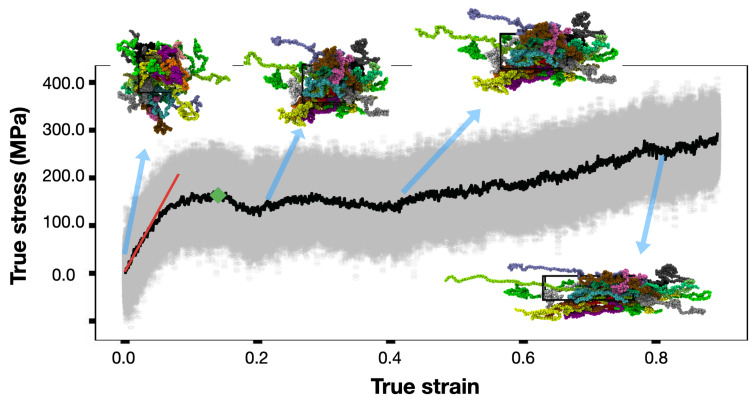
Stress–strain profile obtained from the deformation simulations along with the representative snapshots of an amorphous polymer box illustrating the deformation process at different strain values. The polymer box consists of 10 P3HB chains, each with 500 monomer units. Deformation simulations were performed at 300 K by applying a constant strain rate of 1 × 10^9^ s^−1^. The grey dots represent the instantaneous values and the black line represents the moving average obtained over a window of 1000 points. The red line represents the linear fit in the small-strain regime used to determine Young’s modulus. Yield stress is identified by the green diamond. Representative snap shots of the simulated polymer structure at four different strains are shown, where each chain in the structure is colored separately for clarity.

To determine the mechanical properties, amorphous polymer structures generated using EMC [40] were geometry optimized, followed by a 1 ns MD run at 700 K for thermal equilibration in the NVT ensemble. Subsequently, the structures were quenched by uniformly ramping down the temperature from 700 K to 300 K in 2 ns and further equilibrated at 300 K for 10 ns. Deformation simulations were performed by applying a strain at the constant rate along the longitudinal direction, while the pressure in the other two transverse directions was maintained by the barostat allowing the simulation box to relax in these dimensions. Stress and strain values were computed and stored for every time-step throughout the simulation trajectory. The inset to Figure 1 shows representative snapshots of the simulation cell illustrating the deformation process. VMD [46] was used for the visualization of MD trajectories and rendering the snapshots. A typical stress–strain profile obtained from the simulation trajectory is shown in Figure 1. Transparent gray points represent the instantaneous stress values with large fluctuations. A moving average was obtained in order to smoothen out the stress–strain profile for determining the mechanical properties, i.e., Young’s modulus and yield stress. For the former two, only the initial deformation responses pertaining to the low strain values (up to 3%) were considered, consistent with the formalism adopted in previous studies [47,48]. A linear fit to the stress–strain profile in the low-strain regime was determined, as represented by the red line in Figure 1 and slope of the line gives the Young’s modulus. Poisson’s ratio was determined by plotting the strain in the longitudinal direction vs. the average strain along the other two directions (perpendicular to the applied strain direction). Once again, a linear fit was obtained in the small-strain regime (below 3%) and the slope of this fit gives the Poisson’s ratio. Yield point, represented by the green diamond in Figure 1, is identified as the maximum stress in the elastic regime or where the elastic region ends. Beyond the yield point, stress decreased with increase in strain due to strain-softening. Once the stress reached the local minimum, the stress again increased with applied strain. This phenomena is known as strain-hardening [34,49,50].

To estimate the underlying uncertainty in the computed property, a total of five independent amorphous polymer structures were generated by changing the random seed in the EMC [40] polymer generator. For each system, deformation simulations were performed by applying strain along the three Cartesian directions, independently. Thus, all the reported mechanical properties of the polymers simulated in this work were averaged over 15 instances (5 different configurations and 3 different directions for each).

## 3. Results

### 3.1. Effects of Strain Rates and Temperature

**Figure 2:**
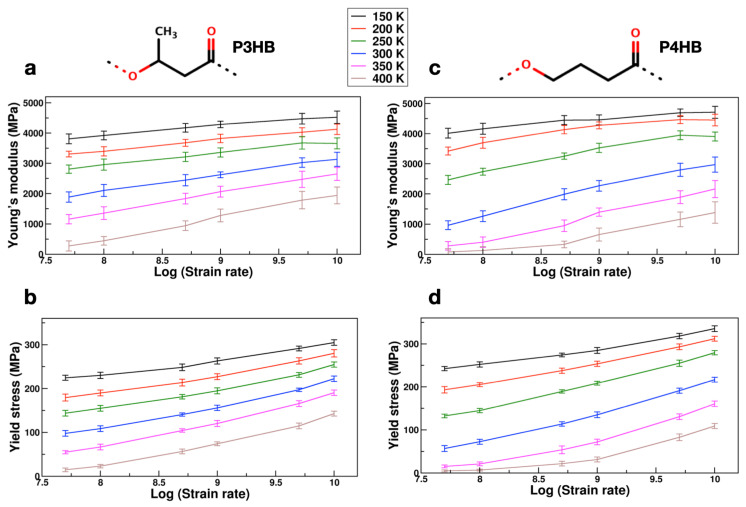
Variation of Young’s modulus and yield stress values (in MPa) with temperature and applied strain rates. Two polymer chemistries P3HB and P4HB were considered for this study and their chemical structures shown in the top panel. (**a**,**b**) Young’s modulus and yield stress values for P3HB; (**c**,**d**) Young’s modulus and yield stress values for P4HB. For each data set, a total of 15 simulations were performed using 5 independently generated amorphous polymer structures and applying strain in 3 different directions for each amorphous polymer cell.

Mechanical properties of polymers are known to depend on the applied strain rate [47,51] as well as temperature [48,52]. Herein, we have studied the mechanical response of two prototypical PHAs, P3HB and poly(4-hydroxybutyrate) (P4HB) by varying both the temperature and strain rates. The simulated temperatures were 150, 200, 250, 300, 350, and 400 (in K) and the applied strain rates were 1 × 10^10^, 5 × 10^9^, 1 × 10^9^, 5 × 10^8^, 1 × 10^8^, and 5 × 10^7^ (in s^−1^). As mentioned in the previous section, a total of 15 simulations (5 independent configurations; 3 different directions) were carried out at each temperature and strain rate value for a better statistical average and standard deviation. Figure 2 shows the Young’s modulus and yield stress values obtained as a function of various temperatures and strain rates for P3HB and P4HB, respectively. These results suggest that as the strain rate decreases, Young’s modulus and yield stress also decrease systematically; while a similar behavior can be achieved by increasing the temperature. These findings are consistent with previously reported work by Sahputra et al. [48]. The profiles for Poisson’s ratio were provided in Figure S2 of the Supplementary Information. Poisson’s ratio increased with an increase in temperature and similar trends were observed in the literature for various polymer systems [53,54].

We note here that a direct comparison of the simulation results with the experiments is practically hindered since the magnitude of strain rate in simulations are generally several orders of magnitude higher compared those used in measurements. Accessing these low strain rates in simulations can be extremely challenging due to large computational time and resource requirements [48]. However, we note that the relative trends obtained in the mechanical properties at high strain rates in simulations are expected to correlate well with the corresponding experimental measurements [34,36,55]. For instance, Figure S3 of Supplementary Materials shows the correlation between the Young’s modulus determined from our simulations and experimental measurements (including our in-house experimental results) for P3HB and P4HB [56].

Lastly, we note that more sophisticated hybrid molecular mechanics–molecular dynamics (MM–MD) methods can be employed to study mechanical properties of polymers at various temperatures. For instance, a method recently proposed by Sahputra et al. [57] used MM, energy minimization, and MD in synergy to first drive the system to the stable state at the target temperature where the macroscopic mechanical properties were calculated. While such an approach can overcome the limitation that MD is restricted to extremely high strain rates as compared to experiments, it does require a substantially large amount of computational resources (about a factor of 10 larger than the present approach). Given that we are mainly interested in understanding relative changes in the mechanical properties as a function of chemistry, we adopted the traditional approach which has been used widely by others [34,36,48,58,59,60,61].

### 3.2. Trends in Mechanical Properties of Homopolymers

**Figure 3:**
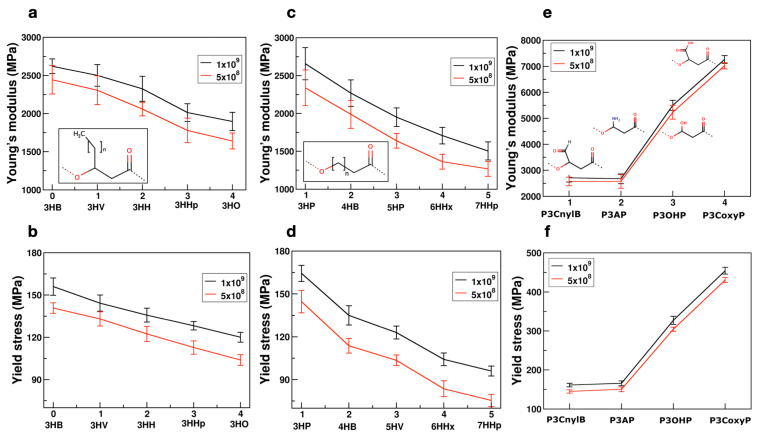
Comparison of Young’s modulus and yield stress values (in MPa) for PHAs with different side chain carbon length (**a**,**b**), backbone carbon length (**c**,**d**), and side chain functional group (**e**,**f**), respectively. Chemical structures of polymers considered in this study are shown in the inset. For each polymer, simulations were performed at 300 K by applying two different strain rates and averaged over 15 independent runs.

After a careful evaluation of the mechanical response as a function of temperature and strain rate, as a next step we performed simulations with the identified strain rate at the room temperature to study relative trends across different PHA polymer chemistries. We reiterate that while our computational results are expected to significantly overestimate the mechanical strength due to applied high strain rates, the relative qualitative trends with respect to systematic changes in the chemical structure of the polymer (number of carbons in the backbone, side chain length and functional groups etc.) are expected to be correctly captured in our simulations, which can provide crucial information towards the rational design of biodegradable polymers for targeted applications. As before, for each polymer system, a total of 15 simulations, averaged over 5 independently-generated amorphous structures and 3 different directions, were simulated. Figure 3 shows the mean and standard deviation values obtained using the 15 independent simulations carried out at 300 K.

Figure 3a,b displays the variation of Young’s modulus and yield stress, respectively, for PHAs with different side chain alkyl groups. The inset to the figure shows the chemical structure of PHA molecules whose carbon chain was varied from methyl to pentyl (n = 0 to 4). As the length of carbon chain increased, the Young’s modulus and yield stress decreased for both the strain rates. This is consistent with the behavior observed in the case of poly(3-alkylthiophenes) polymers [62]. Additionally, in our previous work [29], we have shown that the glass transition temperature ($T_{g}$) decreases with increasing side chain length; and therefore, at 300 K P3HO polymers appear to be much more flexible than the P3HB chains. As the side chain length is increased, both $T_{g}$ and mechanical strength decreased, which is consistent with trends in the literature [47,63,64]. We also note that softening of the polymer structure decreases the mechanical strength, and the elongation at break increases, which is a critical property that directly influences the processability of the polymer for a wide range of applications, including packaging applications [65].

Figure 3c,d shows the mechanical properties of PHAs with a varying number of carbon atoms in the backbone. Chemical structures of the PHAs studied are shown in the inset with the number of carbon atoms ‘n’ systematically changing from 1 to 5. These results indicate that the mechanical strength (i.e., Young’s modulus and yield stress) decreases with increasing carbons in the backbone. The chemical trends obtained in our simulations are in qualitative agreement with experimental observations by Meng et al. [56], who reported that going from 3HP to 4HB leads to a significant decrease in the Young’s modulus. Additionally, the experimental $T_{g}$ values for 3HP and 4HB are 255.3 K and 226.15 K, respectively [56], suggesting that at the room temperature the 4HB chains are much softer than that of 3HP leading to a significant decrease of Young’s modulus with an additional carbon atom in the polymer backbone. The simulation results are also in agreement with what has been reported for other polymer classes, for instance, amidoamines [66]. Overall, with the increase in the number of carbon atoms either in the side chain or in the polymer backbone, the Young’s modulus and yield strength decreases. However, the rate of decrease in the case of the backbone is higher compared to that of the side chain. Figure S4 of Supplementary Materials also shows the comparison between the mechanical properties of PHAs with different side chains and backbones, normalized with respect to the PHA with the smallest alkyl chain. Combining the effects of side chain and backbone, PHA chemical space offers more tunability in terms of designing polymers with specific mechanical properties. For example, the alkyl chain length can be varied simultaneously both in the side chain and backbone to further tailor other functional properties, and we anticipate the PHAs to exhibit the mechanical properties in between the trends of Figure 3a–d.

In addition, we have also performed simulations to explore the mechanical properties by changing the side chain functional group. Specifically, we studied carbonyl (-CHO), amine (-NH_2_) hydroxyl (-OH), and carboxyl (-COOH) groups. The chemical structures of the studied polymers are provided as the inset to the Figure 3e. The Young’s modulus and yield stress values shown in Figure 3e,f clearly indicate that there is a strong correlation with respect to the chemical nature of the side chain functional group. In our earlier work [29], we have shown that there is a strong dependence of $T_{g}$ on the chemical nature of the side chain functional group and it is highly correlated to the net absolute partial charge on the side chain functional group. In this study, we confirm a strong correlation between the group polarity and mechanical properties as shown in Figure S5 of Supplementary Materials. The predicted trends can be qualitatively rationalized based on strengthening the interchain interactions due to the polar side chain groups. For example, PHAs with carboxyl groups can exhibit inter chain hydrogen bonds [29] that significantly enhance the inter chain interactions.

### 3.3. Mechanical Properties of Binary and Ternary Copolymers

**Figure 4:**
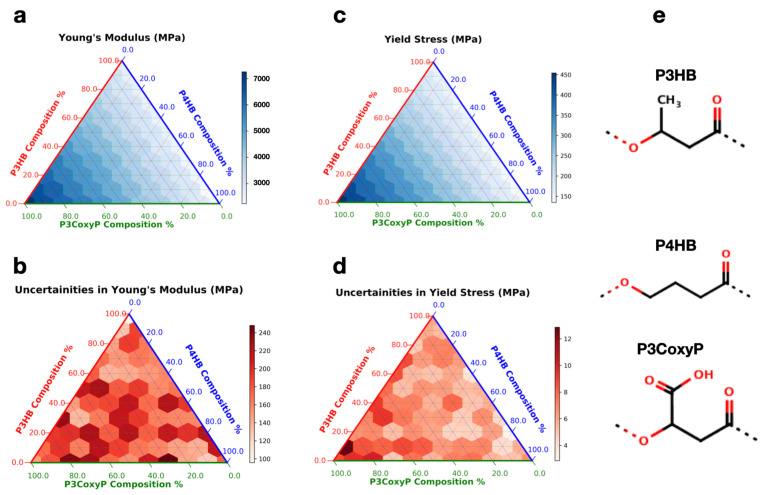
Mechanical properties and associated uncertainties (in MPa) of a ternary polymer P4HB–P3HB–P3CoxyP: (**a**,**c**) represent the ternary plots of Young’s modulus and yield stress, while the associated uncertainties are shown in (**b**,**d**). The chemical structures of the polymers are displayed in (**e**). Simulations were conducted at 300 K applying strain of 1 × 10^9^ s^−1^. At each composition, a total of 15 independent simulations were performed to determine the mean and associated uncertainties.

The chemical design space can be substantially increased going from homopolymers to copolymers by combining two or three monomers with different compositions and configurations. In our previous work [30], we have systematically explored the composition- and configuration- dependence of $T_{g}$ in binary copolymers and blends of PHAs. Predictions from simulations were well in agreement with the estimates obtained using the analytical Fox Equation [67]. Such an analytical equation is quite useful to quickly interpret the $T_{g}$ of the copolymer. However, to the best of our knowledge, there is no analytical expression to estimate the mechanical properties of polymer mixtures. Therefore, here we explore atomistic simulations to directly address the compositional-dependence of mechanical properties in multidimensional PHA compositional space by performing simulations over an entire compositional range of P4HB–P3HB–P3CoxyP ternary copolymers. These three polymers are chosen due to their diverse chemistries; while P4HB exhibits no side chains, P3HB has a methyl side chain functional group and P3CoxyP displays carboxyl group as a side chain that can significantly enhance the inter chain interactions due to hydrogen bond formation. Multi-component amorphous polymers were generated with random configurations using the EMC package [40] for every 10 mol% composition. In each chain, monomers of different types were randomly distributed along the polymer backbone and no two chains in the amorphous box were identical to each other. All the simulations were performed at 300 K by applying strain at a rate of 1 × 10^9^ s^−1^. Figure 4 represents a ternary plot of Young’s modulus and yield stress values (top panel) averaged over 15 independent simulations along with their uncertainties (bottom panel). Here, three corners represent the homopolymers, and three edges represent binary copolymers of P4HB–P3HB, P4HB–P3CoxyP, and P3HB–P3CoxyP. The mean values of the properties and their uncertainties are shown in white-blue and white-red color bars, respectively. Table S1 of Supplementary Materials shows the corresponding tabular data of these ternary plots.

As a general trend, it can be seen from Figure 4 that both the mechanical properties vary smoothly as a function of composition and the associated uncertainties are relatively low (i.e., 3% to 4%) across the entire chemical space. Further, as the P3CoxyP composition increases, both the Young’s modulus and yield stress increase. This can be rationalized based on enhanced inter chain interactions due to hydrogen bond formation leading to a significant strengthening of the copolymer at larger P3CoxyP compositions. This information, along with structure–property relationships is crucial for training data-driven models and predicting new polymer chemistries with desired properties of interest.

## 4. Discussion

Overall the results presented above indicate that our simulation approach can capture trends in the target mechanical properties as a function of temperature, strain rate, as well as systematic variations in the structural and chemical aspects of the motifs forming the polymer in a physically meaningful manner. To start with we systematically evaluated temperature and strain rate dependence of the mechanical response for prototypical PHAs, such as, P3HB and P4HB. We find that while simulations at high strain rates (such as 1 × 10^10^ s^−1^) are computationally efficient, the associated results generally have relatively larger error bars in the properties due to smaller simulation times. On the other hand, computations with smaller strain rates are much more expensive and can be practically challenging for a wide range of PHA chemistries. Therefore, to achieve the best trade-offs between the accuracy and computational cost, our subsequent simulations were performed at the strain rates of 1 × 10^9^ s^−1^ and 5 × 10^8^ s^−1^ at the room temperature to systematically study the influence of polymer structure (side chain length, backbone length, and the side chain functional group) and composition on the mechanical properties. Our results indicate that while a larger number of carbon atoms in both side chain and backbone lead to softening of PHAs (translating to a lower Young’s modulus and yield stress), the sensitivity of the mechanical properties with respect to the number of carbon atoms in the backbone was much higher. In addition, the mechanical strength also showed a strong dependence on the chemical nature of the side chain functional groups via altering the relative inter-chain interactions. Finally, with a specific example of P4HB–P3HB–P3CoxyP ternary copolymer, we demonstrated how the developed computational approach can be used to study systematic chemical trends across multi-component PHA chemistries to study relative chemical trends as a function of composition. Overall, our simulations bring forward interesting trends of structure–property relationships and compositional dependence of mechanical properties in PHA-based polymers. Our results and the approach can potentially be useful to accelerate the design and development of new polymers with targeted properties.

## Data Availability

The data presented in this study are available on reasonable request from the corresponding author.

## Supplementary Materials

The following are available online at https://www.mdpi.com/article/10.3390/polym14020345/s1, Figure S1: Two-dimensional chemical structures of building blocks of PHA-based monomers that were considered in this study.;Figure S2: Variation of Poisson’s ratio with temperature and applied strain rates for (a) P3HB and (b) P4HB. The chemical structures are shown in the top panel. For each data set, a total of 15 simulations were performed using 5 independently generated amorphous polymer structures and applying strain in 3 different directions for each amorphous polymer cell; Figure S3: Variation of Young’s modulus for P3HB and P4HB at two different magnitude of strain rates. The open and closed symbols represent the Young’s modulus values determined using simulations and measured in experiments, respectively. Figure S4: Normalized mechanical properties of PHA-based polymers with respect to the corresponding PHA with the smallest alkyl chain. (a) Young’s modulus, (b) Yield stress. Solid and dashed lines represent the properties of PHAs with varying side chain and backbone length, respectively. X-axis denotes the PHAs with different side chain length and the one in parentheses represents PHA with different backbone length. Figure S5: Comparison of (a) Young’s modulus and (b) yield stress values (in MPa) for PHAs as a function of net absolute partial charge on the side chain functional group. Table S1: Mechanical properties, Young’s modulus and yield stress of a ternary polymer P4HB–P3HB–P3CoxyP. All the values are in MPa. Simulations were conducted at 300 K applying strain of 1 × 10^9^ s^−1^. At each composition, a total of 15 independent simulations were performed. Values in the parentheses are associated uncertainties.
