# Supplementary material for: Predicting the Mechanical Response of Polyhydroxyalkanoate Biopolymers Using Molecular Dynamics Simulations
Source: Polymers (Basel). 2022 Jan 17;14(2):345. doi: 10.3390/polym14020345 (PMC8778129; doi:10.3390/polym14020345)
Supplement: Supplementary file 1 [file polymers-14-00345-s001.zip › polymers-1527945-supplementary.pdf]

Supporting Information for: Predicting the Mechanical Response of Polyhydroxyalkanoate Biopolymers Using Molecular Dynamics Simulations

Karteek K. Bejagam,[*,†] Nevin S. Gupta,[‡] Kwan-Soo Lee,[‡] Carl N. Iverson,[‡] Babetta L. Marrone,[¶] and Ghanshyam Pilania[*,†]

†*Materials Science and Technology Division, Los Alamos National Laboratory, Los Alamos, NM 87545, USA*

‡*Chemistry Division, Los Alamos National Laboratory, Los Alamos, NM 87545, USA*

¶*Bioscience Division, Los Alamos National Laboratory, Los Alamos, NM 87545, USA*

E-mail: karteekbeja@lanl.gov; gpilania@lanl.gov

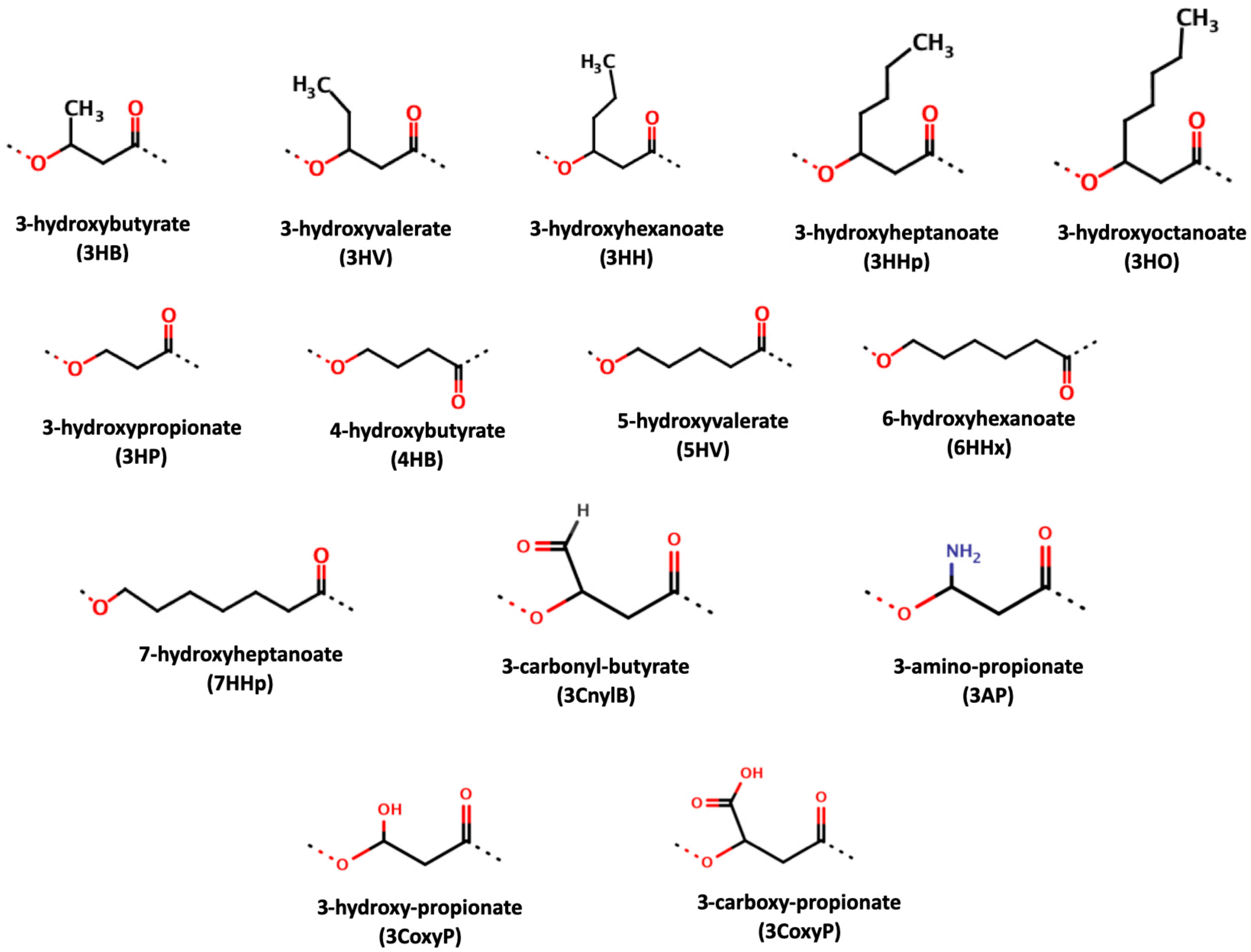

Figure S1: **Two-dimensional chemical structures of building blocks of PHA-based monomers that were considered in this study.**

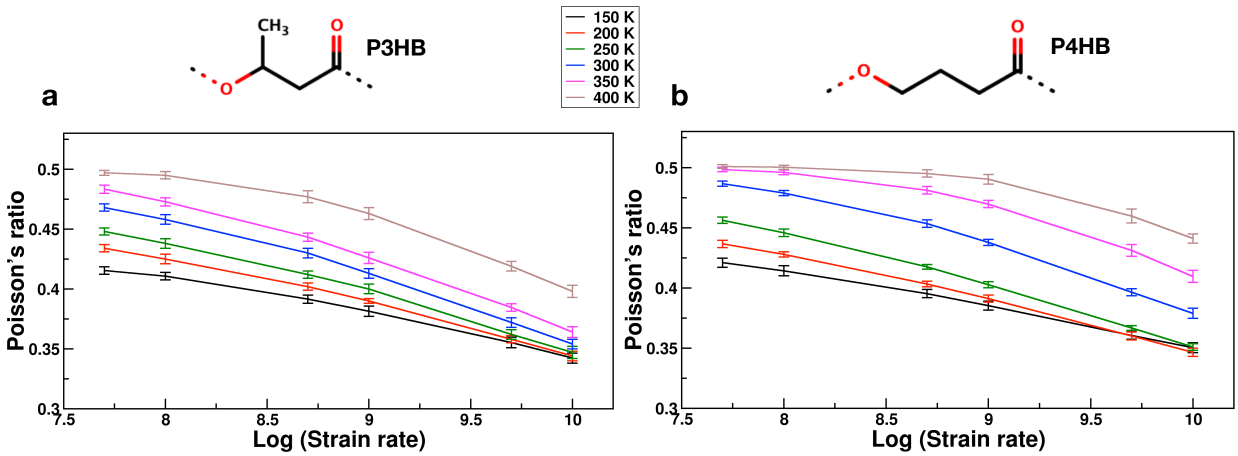

Figure S2: Variation of Poisson's ratio with temperature and applied strain rates for (a) P3HB and (b) P4HB. The chemical structures are shown in the top panel. For each data set, a total of 15 simulations were performed using 5 independently generated amorphous polymer structures and applying strain in 3 different directions for each amorphous polymer cell.

1 Details of Experimental Setup

P3HB granule (BU396312) films were purchased from Goodfellow (Coraopolis, PA, USA). HPLC grade chloroform from Fisher Scientific (Walham, MA, USA) was used to dissolve the P3HB granules during film casting. For P3HB preparation, an approximately 2.5% (w/w) solution of P3HB in chloroform was prepared by constant mixing at 50 ± 5°C. The solution was then casted on a glass substrate and the chloroform was allowed to evaporate at room temperature. The resulting film was dried for at least 2 hours under vacuum to remove residual chloroform. P3HB samples (12.7 mm by 102 mm) with a thickness of 35 ± 2 μm are used for tension testing. Samples were run using an Instron 3343 Single Colum Testing System (Norwood, MA, USA). The samples were placed so there was a 45 ± 5 mm gauge length (distance between sample grips) and then pulled to break with the appropriate rate. Bluehill Universal Software was used to analyze the data.

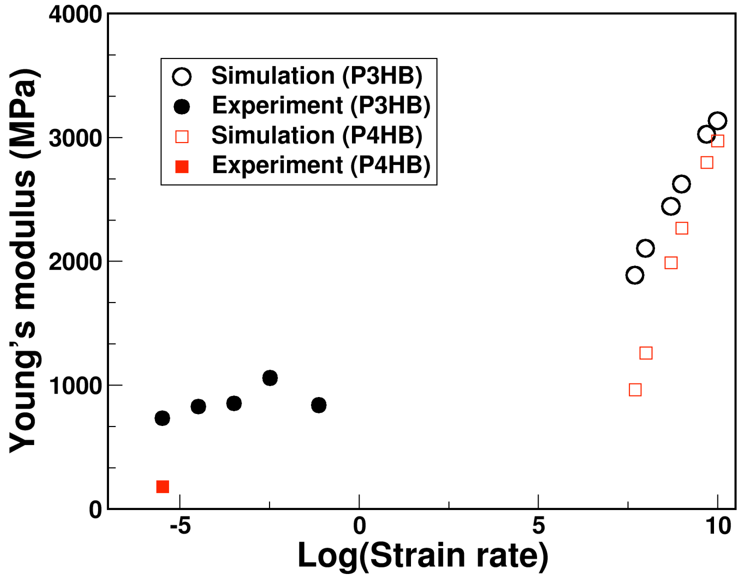

Figure S3: Variation of Young's modulus for P3HB and P4HB at two different magnitude of strain rates. The open and closed symbols represent the Young's modulus values determined using simulations and measured in experiments, respectively.

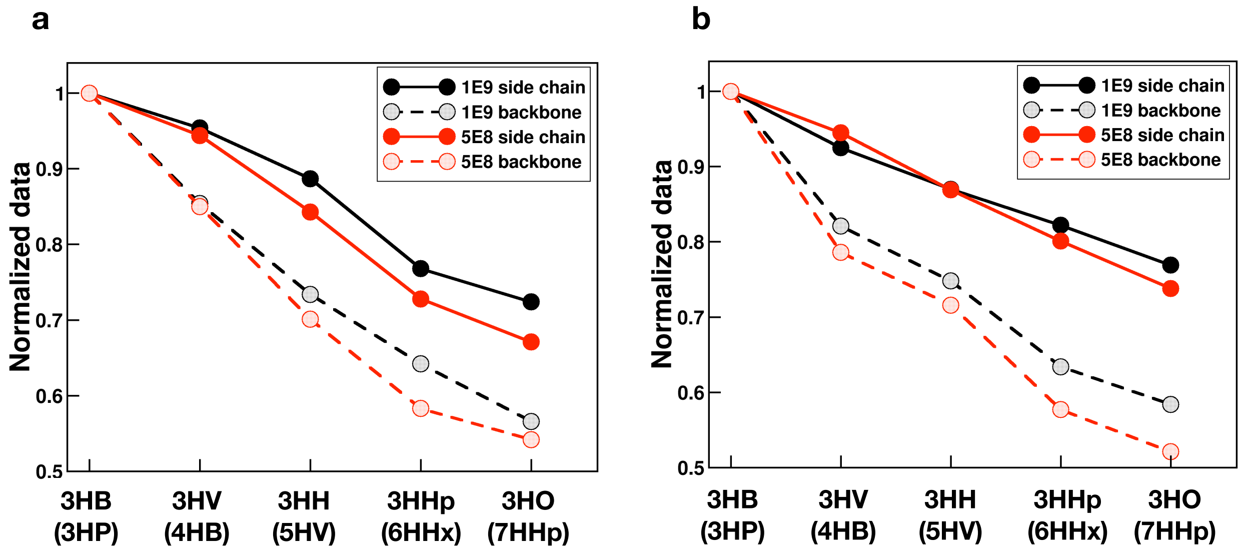

Figure S4: Normalized mechanical properties of PHA-based polymers with respect to the corresponding PHA with the smallest alkyl chain. (a) Young's modulus, (b) Yield stress. Solid and dashed lines represent the properties of PHAs with varying side chain and backbone length, respectively. X-axis denotes the PHAs with different side chain length and the one in parentheses represents PHA with different backbone length.

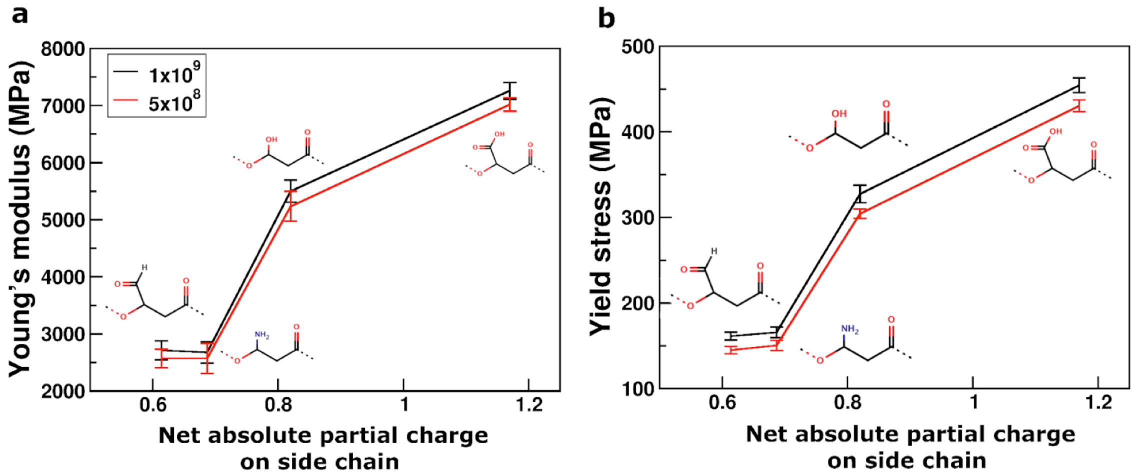

Figure S5: **Comparison of (a) Young's modulus and (b) yield stress values (in MPa) for PHAs as a function of net absolute partial charge on the side chain functional group.**

Table S1: Mechanical properties, Young's modulus and yield stress of a ternary polymer P4HB–P3HB–P3CoxyP. All the values are in MPa. Simulations were conducted at 300 K applying strain of 1×10^9 s^{-1}. At each composition, a total of 15 independent simulations were performed. Values in the parentheses are associated uncertainties.

Compositions (mol%)			Mechanical properties	
P3HB	P4HB	P3CoxyP	Young's modulus	Yield stress
10	90	0	2653.2(187.9)	156.4(5.3)
20	80	0	2665.0(164.5)	154.1(7.0)
30	70	0	2622.1(157.2)	150.5(6.3)
40	60	0	2546.0(157.3)	151.4(3.8)
50	50	0	2632.7(166.1)	148.9(5.5)
60	40	0	2460.6(193.9)	146.3(5.7)
70	30	0	2433.7(157.0)	144.3(4.3)
80	20	0	2443.1(220.2)	143.0(2.8)
90	10	0	2229.3(213.4)	138.0(5.7)
10	0	90	6637.0(181.8)	419.5(9.5)
20	0	80	6158.9(238.9)	381.6(10.1)

30	0	70	5734.1(229.1)	352.6(4.9)
40	0	60	5312.1(172.0)	323.2(7.6)
50	0	50	4736.3(153.7)	290.0(5.5)
60	0	40	4141.1(209.2)	261.7(7.2)
70	0	30	3885.3(248.2)	231.1(7.6)
80	0	20	3326.4(143.0)	203.4(5.5)
90	0	10	2862.6(124.3)	169.5(5.0)
0	10	90	6595.1(171.8)	419.5(12.9)
0	20	80	6109.4(193.9)	383.9(6.6)
0	30	70	5725.1(232.4)	354.6(7.0)
0	40	60	5185.7(147.8)	319.4(9.1)
0	50	50	4681.3(213.1)	288.5(7.7)
0	60	40	4258.9(178.3)	254.2(8.6)
0	70	30	3940.4(168.9)	230.2(8.6)
0	80	20	3547.9(145.3)	205.9(4.9)
0	90	10	3185.7(128.5)	182.2(5.9)
10	10	80	6248.6(195.7)	383.1(8.5)
10	20	70	5655.8(198.1)	351.1(6.8)
10	30	60	5274.0(134.5)	324.4(9.3)
10	40	50	4724.7(180.9)	291.0(7.1)
10	50	40	4386.5(225.1)	261.4(5.3)
10	60	30	3959.7(137.6)	232.7(5.4)
10	70	20	3511.4(150.8)	205.6(6.3)
10	80	10	3079.2(190.8)	177.6(7.0)
20	10	70	5666.3(162.5)	355.4(8.0)
20	20	60	5246.5(223.8)	322.6(5.3)
20	30	50	4816.6(164.2)	290.6(7.4)

20	40	40	4376.2(186.3)	260.4(7.1)
20	50	30	3881.9(178.2)	234.6(6.5)
20	60	20	3519.2(142.0)	205.2(5.1)
20	70	10	3102.8(164.8)	178.4(7.1)
30	10	60	5200.3(217.9)	323.4(8.7)
30	20	50	4817.7(177.5)	291.3(6.7)
30	30	40	4412.3(213.4)	265.1(6.2)
30	40	30	3951.7(215.4)	232.6(5.6)
30	50	20	3452.4(191.9)	206.2(6.3)
30	60	10	3041.9(150.4)	180.5(5.9)
40	10	50	4746.8(190.3)	295.6(8.2)
40	20	40	4300.7(220.3)	263.4(6.0)
40	30	30	3880.9(193.3)	232.4(6.4)
40	40	20	3555.6(171.3)	204.6(7.9)
40	50	10	3015.6(156.0)	177.9(5.3)
50	10	40	4350.1(165.8)	262.7(6.4)
50	20	30	3878.0(176.6)	233.7(4.6)
50	30	20	3423.1(162.0)	203.3(4.7)
50	40	10	2955.5(221.1)	176.5(5.0)
60	10	30	3849.9(158.7)	236.5(6.9)
60	20	20	3478.1(212.8)	207.0(4.6)
60	30	10	3033.0(157.7)	178.5(6.7)
70	10	20	3416.1(146.2)	201.6(6.9)
70	20	10	2989.0(195.8)	171.7(6.2)
80	10	10	2962.0(139.5)	171.7(4.2)
0	100	0	2620.7(95.7)	156.0(6.1)
100	0	0	2268.6(175.5)	135.0(6.7)

0	0	100	7261.6(146.9)	454.3(8.6)
